# The Association Between Dietary Fiber Intake and Depression Among US Adults: A Cross‐Sectional Study Based on NHANES Data From 2005 to 2020

**DOI:** 10.1002/fsn3.70605

**Published:** 2025-07-16

**Authors:** Siran Lai, Yuning Zeng, Tianyi Li, Yue Li, Yue An, Xueren Ouyang

**Affiliations:** ^1^ Department of Pediatrics Shunde Women and Children's Hospital of Guangdong Medical University Foshan Guangdong China; ^2^ Institute of Traditional Chinese Medicine Shunde Women and Children's Hospital of Guangdong Medical University Foshan Guangdong China; ^3^ Guangzhou University of Chinese Medicine Guangzhou Guangdong China

**Keywords:** CRD, CSD, depression, dietary fiber, NHANES

## Abstract

Depression is a prevalent chronic condition that affects a person's thoughts, emotions, and physical health. However, there is limited evidence on the impact of dietary fiber on depression. Additionally, the association between dietary fiber intake and the risk of depression remains controversial. We extracted data from the National Health and Nutrition Examination Survey (NHANES) database on 85,750 participants. After excluding minors and pregnant individuals, and those with missing Patient Health Questionnaire‐9 (PHQ‐9) data, dietary fiber intake, or covariates, we included 29,980 participants for data analysis. Categorizing them into non‐depressed groups, clinically relevant depression (CRD, with scores no less than 10) and clinically significant depression (CSD, with scores no less than 15) is determined based on PHQ‐9 scores. We employed survey‐weighted generalized linear models, and restricted cubic spline (RCS) curves are employed to judge the significance of the correlation of dietary fiber intake and risk of developing CRD and CSD. Finally, we conducted subgroup analyses using stratified weighted multiple regression analysis. The manuscript was organized according to STROBE guidelines. There exists a non‐linear inverse relationship between dietary fiber intake and the incidence of CRD and CSD. In the model after full adjustment, compared to individuals in the first quartile, the probability of CRD was reduced by 17%, 22%, and 30% respectively for those in the second, third, and fourth quartiles (odds ratios [OR] = 0.70; 95% confidence interval [95% CI]: 0.57–0.85, *p* < 0.001); the risk of CSD was reduced by 22%, 40%, and 32% (OR = 0.68; 95% CI: 0.48–0.94, *p* = 0.02). RCS curves indicate an L‐shaped nonlinear connection existing between dietary fiber consumption and both CRD and CSD. Subgroup analysis strengthens the stability of the conclusions. There is a nonlinear negative relationship of dietary fiber intake and the risk of CRD and CSD, with a nonlinear L‐shaped relationship between dietary fiber intake and both CRD and CSD.

## Graphical abstract

We surveyed the intake of dietary fiber and depressive manifestations of 85,750 individuals, discovered that there is a nonlinear negative relationship of dietary fiber intake and the risk of CRD and CSD, with a nonlinear L‐shaped relationship between dietary fiber intake and both CRD and CSD.

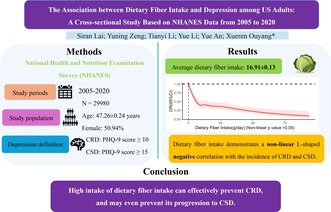

**Abbreviations**
95% CI: 95% confidence interval
BMI: body mass index
CRD: clinically relevant depression
CSD: clinically significant depression
FPED: Food Patterns Equivalents Database
IL‐6: interleukin‐6
MEC: Mobile Examination Center
NHANES: National Health and Nutrition Examination Survey
OR: odds ratios
PHQ‐9: patient health Questionnaire‐9
PIR: poverty income ratio
RCS: restricted cubic spline
SCFA: short‐chain fatty acids
SE: standard errors
STROBE: Strengthening the Reporting of Observational Studies in Epidemiology
TNF‐α: tumor necrosis factor‐alpha

## 1 Introduction

Depression is a prevalent chronic condition affecting thinking, emotions, and bodily health, marked by a downcast mood, deficiency of energy, sorrow, sleeplessness, and an incapacity to relish life (Cui 2015). Epidemiological data from 2019 indicated that major depressive disorder was the second leading cause of disability burden worldwide when assessed through Years Lived with Disability metrics among the top 25 global health concerns (Ferrari et al. 2022). The post‐COVID‐19 era has witnessed a marked increase in the incidence and clinical severity of major depressive disorder. The Coronavirus Disease 2019 pandemic contributed to a global surge of approximately 53.2 million additional cases of major depressive disorder, reflecting a 276% increase and elevating the prevalence rate to 3152.9 cases per 100,000 population (Santomauro et al. 2021). The chronic nature of depression, which can persist for many years, further exacerbates its impact on individuals and societies (Smith 2014). Considering the significant societal and individual toll of depression, pinpointing its risk factors is crucial for public health efforts.

As per the definition provided by the Codex Alimentarius Commission in 2009, dietary fiber refers to carbohydrate (CHO) polymers that consist of 10 or more monomeric units and are not subject to hydrolysis by the endogenous enzymes present in the human small intestine. It includes edible CHO polymers naturally found in food as consumed, those derived from food raw materials through physical, enzymatic, or chemical processes, and synthetic CHO polymers (Jones 2014). To our knowledge, dietary fiber is connected with intestinal disease. However, a growing body of research indicates that dietary fiber may also be connected with depression. A meta‐analysis covering different countries, including the US, Europe, Asia, and Australia, revealed that a high amount of dietary fiber among adolescents and adults is substantially connected with a reduced likelihood of depression. According to the analysis, for every additional 5 g of dietary fiber consumed, the risk of developing depression in adults decreases by 5% (Saghafian et al. 2023). Fatahi et al. (2021) discovered through a comprehensive systematic review and meta‐analysis of observational studies that increasing consumption of dietary fiber is capable of reducing developing depression risk. A study shows that young people with elevated depressive symptoms who participate in and adhere to dietary interventions, specifically a healthy diet including fruits, vegetables, fish, and lean meats, can alleviate their depressive symptoms. Although the study did not fully exclude concurrent pharmacological or psychotherapeutic treatments, it employed randomized allocation and covariate adjustments to establish that dietary interventions retain independent efficacy after controlling for other variables (Francis et al. 2019). A study focusing mainly on middle‐aged and elderly people indicates that a dietary pattern characterized by high intake of butter, chocolate, candy, added sugars, high‐fat cheeses, and low intake of fresh vegetables and fruits can lead to an increase in symptoms of depression and anxiety (Chen, Cao, et al. 2023). However, some studies have reported divergent findings. Li et al. (2020) proposed that, in early perimenopausal women, there's no significant link between dietary fiber intake and depressive symptoms. Similarly, a cross‐sectional study of Japanese workers also found no significant link between total dietary fiber intake and depressive symptoms (Miki et al. 2016). Differences in research findings on the association between dietary fiber and depression may stem from variations in study design, methodological differences, population heterogeneity, the complexity of biological mechanisms, as well as differences in dietary assessment methods and control of confounding factors. Overall, the association between dietary fiber intake and depression remains controversial. Additionally, most current studies have focused on specific populations and singular depressive phenotypes. Epidemiological evidence from large‐scale general populations remains limited, and there is a lack of research distinguishing between clinically relevant depression (CRD) and clinically significant depression (CSD). Data for this study were derived from the National Health and Nutrition Examination Survey (NHANES), a database that ensures national representation of the health and nutritional status of the US non‐institutionalized population through its unique stratified four‐stage probability sampling design and composite weighting correction techniques (Terry et al. 2024).

Therefore, this study utilized the NHANES databases spanning from 2005 to 2020, investigating the potential association between dietary fiber intake and both CRD and CSD. We hypothesized that higher dietary fiber intake would be inversely associated with depression risk, such that increased consumption of dietary fiber correlates with a reduced likelihood of depression.

## 2 Materials and Methods

### 2.1 Overview of NHANES

The data utilized in this study originates from NHANES, a thorough dataset on health and nutrition managed by the national center for health statistics. NHANES has been conducting continuous surveys, collecting health and nutritional status data of a representative sample of around 5000 individuals annually across the United States since 1999. The data sets for each cycle contain multiple types of information, such as data from questionnaires on demographic, socioeconomic, diet, and health‐related questions, as well as content from physical examination components such as physiological measurements and laboratory tests. Approved by the Research Ethics Review Committee of the national center for health statistics, the NHANES program protocols were strictly adhered to the ethical principles set forth in the Declaration of Helsinki.

### 2.2 Study Participants

**FIGURE 1:**
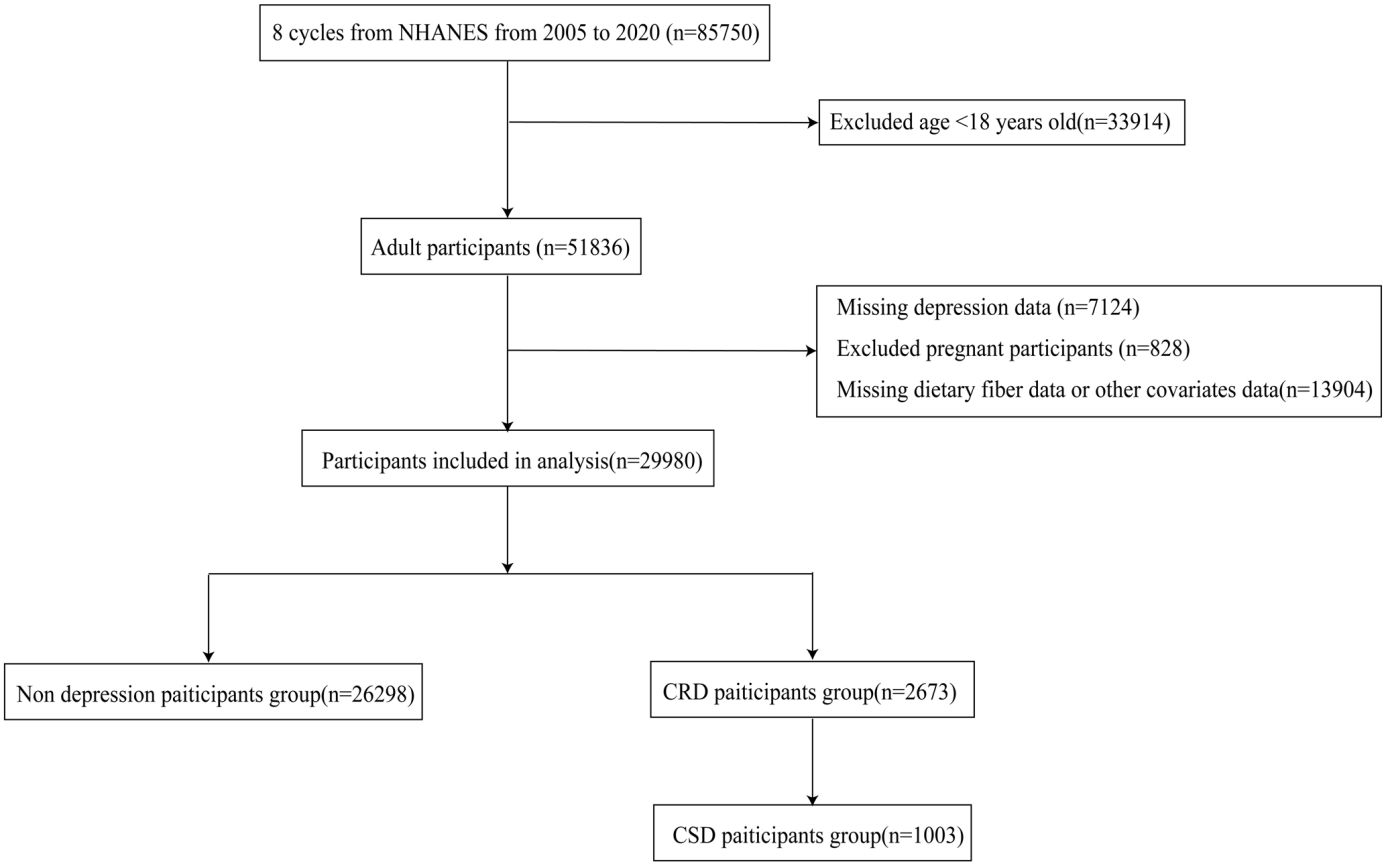
Flowchart of participants selection.

This study employed data spanning eight survey cycles from 2005 to 2020. We evaluated 85,750 participants across these eight consecutive NHANES cycles. This study excluded 33,914 adolescents under 18 and 828 pregnant individuals, as their unique dietary requirements and mental health characteristics might confound the analysis of associations between dietary fiber intake and depression (Yan et al. 2025). The exclusion of individuals under 18 was based on evidence indicating that the presentation of major depressive disorder and depressive symptoms differs between adults and adolescents (Rice et al. 2019). Additionally, the NHANES database provides the Patient Health Questionnaire‐9 (PHQ‐9) questionnaire data only for participants aged 18 and older. Therefore, to maintain consistency and accuracy in our analysis, we excluded participants under 18. Pregnant individuals were excluded due to the unique physiological and psychosocial changes associated with pregnancy (Biaggi et al. 2016), which can significantly influence mental health and potentially confound the relationship between dietary fiber intake and depression. Additionally, records with missing PHQ‐9 questionnaire information (*n* = 7124) and those lacking data on both dietary fiber intake and covariates (*n* = 13,904) were removed. Covariates included demographic characteristics (age, sex, race, education level, body mass index [BMI]), lifestyle factors (marital status, energy intake, work activity, recreational activity, poverty‐income ratio [PIR]), and health conditions (diabetes status, hypertension status, smoking status, alcohol consumption status). Through multi‐stage exclusion criteria, we ensured data integrity for key variables in the study sample, thereby enhancing the validity and reliability of analytical outcomes (Li and Lan 2025). Ultimately, 29,980 participants were encompassed in the study for investigating the cross‐sectional relationship in which the intake of dietary fiber was associated with depression, among whom 2673 individuals were diagnosed with CRD, with further screening identifying 1003 individuals meeting the criteria for CSD. Consent given in writing was provided by all participants and the systematic flowchart for participant selection is depicted in Figure 1. Since the NHANES data set utilized in this study is publicly available, no extra ethical or administrative approvals were necessary.

### 2.3 Assessment of Depression

Since 2005, NHANES has incorporated the PHQ‐9 (Kroenke et al. 2001) to assess signs and symptoms of depression (Liu et al. 2023; Shi et al. 2021; Wu et al. 2023). Within the Mobile Examination Center (MEC), alongside the PHQ‐9 questionnaire, additional computer‐assisted personal interviews are conducted following standardized protocols, along with the collection of anthropometric data such as weight and waist‐to‐height ratio and the gathering of biological samples. The PHQ‐9, a nine‐item instrument, is designed for screening depression (Kroenke et al. 2001). It queries regarding the occurrence frequency of depressive or negative mental manifestations in the past fortnight. The overall score of PHQ‐9 lies within the range of 0 to 27. Cut‐off values classify the severity of depression as “negligible or minimal” (0–4), “slight” (5–9), “moderate” (10–14), “fairly severe” (15–19), and “severe” (20–27). In this study, scores from 10 to 27 (i.e., “moderately severe or severe”) are merged to enhance the precision of estimation. By combining these categories, we increase the sample size within the more severe depression range. This enhances the statistical power of our analysis and allows for more precise estimation of the relationship between dietary fiber intake and depression severity. We designated a total PHQ‐9 score of at least 10 as CRD (Levis et al. 2020), which is in accordance with the performance measure for depression process/outcome recommended by the National Quality Forum. A PHQ‐9 score of at least 10 has demonstrated 88% sensitivity and specificity for major depressive disorder (Kroenke et al. 2001). Additionally, a PHQ‐9 score of at least 15 (a portion of PHQ‐9 scores of at least 10) is classified as CSD (Wang et al. 2016), signifying the existence of main depressive disorder and suggesting active treatment through medication and/or psychotherapy. These cutoffs are widely used and validated in NHANES studies to distinguish between different levels of depression severity (Jia et al. 2019; Wang et al. 2016).

### 2.4 Assessment of Dietary Fiber Intake

Information on dietary intake was employed to calculate the kinds and quantities of eaten items and drinks consumed within the past 24 h (from midnight to the next midnight), thereby estimating the caloric intake, nutrients, and other components derived from these sources. Each NHANES participant underwent two interviews to recall their dietary intake within a 24‐h timeframe. They carried out the first interview at the MEC in person, and the subsequent interview was carried out via telephone 3 to 10 days afterwards. The comprehensive protocol and means of data gathering are thoroughly described in the NHANES Dietary Interview Procedure Manual. The complete methodology is detailed in the NHANES Dietary Interview Procedure Manual (https://wwwn.cdc.gov/nchs/nhanes/continuousnhanes/questionnaires.aspx?Cycle=2019‐2020). We categorized the percentage of dietary fiber intake based on quantiles. Similar to previous studies (Chen, Zhao, et al. 2024; Zhang et al. 2023, 2022), this research selected the dietary fiber (in g) from the total nutrient intake on the first day, derived from the 24‐h initial in‐depth dietary recall interview that was gathered up close and personal at the MEC and adjusted for individual body weight factors. The dietary recall method may be subject to recall bias, as participants' memory of dietary intake can be imperfect. While standardized tools and multiple‐pass 24‐h recalls help minimize this bias, some degree of inaccuracy may still affect the precision of our dietary fiber intake estimates. The sources of dietary fiber, including green groceries and cereals, were identified by consolidating the relevant food categories (Fuller et al. 2016). The total intake of dietary fiber source foods, including fruits, vegetables, and grains, included in this study was obtained from the Food Patterns Equivalents Database (FPED). The FPED converts food from What We Eat in America and NHANES into 37 food pattern components. The FPED serves as a unique research tool to assess the food intake of Americans. The fruit group consists of citrus, melons, berries, other fruits, and fruit juice. The vegetables group consists of dark green, red and orange, starchy, and other vegetables. The grains group consists of whole grains and refined grains.

### 2.5 Covariate Assessment

We conducted a comprehensive screening of 14 potential confounding factors related to depression and identified age, gender, race, education level, BMI, PIR, marital status, energy intake, work activities, recreational activities, diabetes, hypertension status, smoking status, and alcohol consumption as risk factors. This selection ensures that our results are adjusted for variables that could potentially confound the relationship between dietary fiber intake and depression incidence (Zhang et al. 2024). The age span of patients ranged from 20 to 80 years. The genders comprised male and female (Salk et al. 2017). Races consisted of non‐Hispanic White, non‐Hispanic Black, Mexican American, other Hispanic, and other ethnic groups. Education levels were categorized into three groups: below high school, high school, and college or higher. The mean BMI is 29.15 and is computed by calculating the weight in kilograms divided by the square of height in meters. PIR was divided into three groups: less than or equal to 1.0, 1.0 to 3.0, and greater than 3.0. According to the latest NHANES criteria, marital status was taken into account various factors and classified as unmarried, divorced/widowed/separated, and married/living with a partner (Bulloch et al. 2017). Vascular risk factors included hypertension and diabetes (diagnosed by a physician, random blood glucose of at least 11.1 millimoles per liter, glycated hemoglobin of at least 6.5%, fasting blood glucose of at least 7.0 millimoles per liter, 2‐h blood glucose of at least 11.1 millimoles per liter, or use of hypoglycemic medications). Alcohol consumption, weight, and height were recorded at the MEC. Drinkers were identified as individuals who consumed alcohol at least 12 times annually (Boden and Fergusson 2011). Individuals who either had never smoked throughout their life span or had smoked < 100 cigarettes were defined as never smokers (Fluharty et al. 2017).

### 2.6 Statistical Analysis

This study was rigorously designed following the Strengthening the Reporting of Observational Studies in Epidemiology (STROBE) guidelines, with a self‐check form detailed in the Supporting Information (von Elm et al. 2007). All statistical examinations of data were carried out using R language version 4.4.1. We made use of the complex sample weights (Day 1 sample weights) put forward by the Centers for Disease Control and Prevention. The sample weights from the eight consecutive cycles were aggregated according to the NHANES guidelines. In the baseline characteristics table, continuous variables are presented as survey‐weighted means (with standard errors), while categorical variables are detailed with sample sizes (and survey‐weighted percentages). When evaluating the relationship of dietary fiber intake and depression incidence, survey‐weighted generalized linear models were utilized. Dietary fiber intake was modeled as a continuous variable to assess its relationship with the log odds of depression incidence. The primary model was designed as a univariate analysis without adjustments for any covariates, providing a baseline assessment of the relationship between dietary fiber intake and depression incidence. Model 1 introduces demographic variables, controlling for race, BMI, gender, and age, to account for fundamental population characteristics. Building upon this, Model 2 further adjusts for socioeconomic and lifestyle factors, including PIR, marital status, and education level, which are known to influence health outcomes. Finally, Model 3 comprehensively adjusts for all identified confounders, such as energy intake, recreational activities, work activities, diabetes status, hypertension status, smoking status, and alcohol consumption, to ensure a robust evaluation of the association between dietary fiber intake and depression incidence. Subsequently, we employed survey‐weighted generalized linear models to further explore the association between different food groups of dietary fiber sources and depression. Restricted cubic spline (RCS) curves were used to examine the dose–response relationship between depression incidence and dietary fiber intake. Moreover, survey‐weighted multinomial logistic regression and subgroup analyses were performed, treating the consequence of taking dietary fiber in as both a categorical variable (grouped into quartiles based on intake percentages) (Chen, Fu, et al. 2024; Zhang et al. 2023) and a continuous variable. Subgroup analyses were stratified by age, BMI, gender, race, education level, PIR, marriage, smoking status, drinking status, diabetes mellitus, hypertension, recreational activity, and work activity. These variables were selected based on their potential influence on the relationship between dietary fiber intake and depression incidence (Tian et al. 2023). This approach allows us to assess the consistency of the association across diverse subgroups. In this study, all statistical analyses were considered significant at *p* < 0.05.

## 3 Results

### 3.1 Participant Characteristics

This study included 85,750 participants from the NHANES database spanning from 2005 to 2020. After screening, 29,980 subjects were ultimately included in the data analysis. Based on the PHQ‐9 questionnaire assessment, participants were categorized into the non‐depressed group (*n* = 26,298), the CRD group (*n* = 2673), and the CSD group (*n* = 1003). Baseline characteristics of the weighted population were descriptively classified according to the CRD and CSD groups, as detailed in Tables 1 and 2, respectively. The primary age of the participants (standard error, SE) was 47.26 (0.24) years, with a mean daily dietary fiber intake (mean ± SE) of 16.91 (0.13) grams. We separately summarized the baseline characteristics of participants with CRD and CSD. The demographic profiles of CRD and CSD participants were broadly similar, with a higher proportion of females, middle‐aged adults, Non‐Hispanic Whites, individuals with higher educational attainment, high‐income groups, married or cohabiting individuals, those with a history of smoking and alcohol consumption, individuals with hypertension, and those reporting low levels of physical activity and recreational activity. The mean (SE) of dietary fiber intake was 14.34 (0.25) g/day for CRD participants and 14.24 (0.47) g/day for CSD participants, both significantly lower than that of the non‐depression group (*p* < 0.0001). This study identified a weighted prevalence of 8.61% for CRD and 3.20% for CSD. These findings are consistent with previous studies that have found demographic factors such as gender, race, and education to be associated with depression risk (Assari 2017), highlighting the importance of considering these variables in public health strategies for depression prevention and management.

**TABLE 1:** Traits of the study population sorted by CRD.

Characteristics	Overall	Non‐CRD	CRD	*p*
*N*	33,687	30,785	2902	
Age, years	47.26 (0.24)	47.36 (0.25)	46.09 (0.43)	0.01
Body mass index (kg/m^2^)	29.15 (0.08)	29.01 (0.08)	30.72 (0.22)	< 0.0001
Gender, *n* (%)				
Female	16,892 (50.14)	15,066 (48.94)	1826 (62.92)	< 0.0001
Male	16,795 (49.86)	15,719 (51.06)	1076 (37.08)	
Race, *n* (%)				
Non‐Hispanic White	14,754 (43.80)	13,479 (43.78)	1275 (43.94)	0.001
Non‐Hispanic Black	7336 (21.78)	6694 (21.74)	642 (22.02)	
Mexican American	4884 (14.50)	4483 (14.56)	401 (13.82)	
Other race	6713 (19.93)	6129 (19.91)	584 (20.12)	
Education level, *n* (%)				
Less than high school	2891 (8.58)	2532 (8.20)	359 (12.37)	< 0.0001
High school	7509 (22.29)	6652 (21.61)	857 (29.53)	
More than high school	23,287 (69.13)	21,601 (70.17)	1686 (58.10)	
Poverty income ratio, *n* (%)				
≤ 1.0	6765 (20.08)	5703 (18.53)	1062 (36.60)	< 0.0001
1.0–3.0	12,967 (38.49)	12,434 (40.39)	533 (18.37)	
> 3.0	13,955 (41.43)	12,648 (41.08)	1307 (45.04)	
Marriage, *n* (%)				
Divorced/separated/widowed	7366 (21.87)	6390 (20.76)	976 (33.63)	< 0.0001
Married/living with partner	20,129 (59.75)	18,833 (61.18)	1296 (44.66)	
Never married	6192 (18.38)	5562 (18.07)	630 (21.71)	
Drinking status, *n* (%)				
No	9007 (26.74)	8141 (26.44)	866 (29.84)	< 0.001
Yes	24,680 (73.26)	22,644 (73.56)	2036 (70.16)	
Smoking status, *n* (%)				
No	18,519 (54.97)	17,349 (56.36)	1170 (40.32)	< 0.0001
Yes	15,168 (45.03)	13,436 (43.64)	1732 (59.68)	
Diabetes mellitus, *n* (%)				
No	27,517 (81.68)	25,367 (82.40)	2150 (74.09)	< 0.0001
Yes	6170 (18.32)	5418 (17.60)	752 (25.91)	
Hypertension, *n* (%)				
No	19,331 (57.38)	17,911 (58.18)	1420 (48.93)	< 0.0001
Yes	14,356 (42.62)	12,874 (41.82)	1482 (51.07)	
Work activity, *n* (%)				
Vigorous	1229 (4.10)	1101 (4.03)	128 (4.79)	0.15
Moderate	6756 (22.54)	6170 (22.59)	586 (21.92)	
Other	21,995 (73.37)	20,036 (73.37)	1959 (73.29)	
Recreational activity, *n* (%)				
Vigorous	2281 (7.61)	2169 (7.94)	112 (4.19)	< 0.0001
Moderate	7737 (25.81)	7208 (26.40)	529 (19.79)	
Other	19,963 (66.59)	17,931 (65.66)	2032 (76.02)	
Energy intake (kcals/day)	2167.27 (8.16)	2175.59 (8.03)	2071.15 (27.67)	< 0.001
Fiber intake (g/day)	16.91 (0.13)	17.13 (0.13)	14.34 (0.25)	< 0.0001

*Note:* Continuous variables are shown as weighted means with standard errors. Categorical variables are presented as unweighted counts along with weighted percentages.

**TABLE 2:** Traits of the study population sorted by CSD.

Characteristic	Overall	Non‐CSD	CSD	*p*
*N*	33,687	32,608	1079	
Age, years	47.26 (0.24)	47.27 (0.25)	46.76 (0.63)	0.47
Body mass index (kg/m^2^)	29.15 (0.08)	29.09 (0.08)	30.92 (0.32)	< 0.0001
Gender, *n* (%)				
Female	16,892 (50.14)	16,190 (49.65)	702 (65.06)	< 0.0001
Male	16,795 (49.86)	16,418 (50.35)	377 (34.94)	
Race, *n* (%)				
Non‐Hispanic White	14,754 (43.80)	14,265 (43.75)	489 (45.32)	0.07
Non‐Hispanic Black	7336 (21.78)	7116 (21.82)	220 (20.39)	
Mexican American	4884 (14.50)	4739 (14.53)	145 (13.44)	
Other race	6713 (19.93)	6488 (19.90)	225 (20.85)	
Education level, *n* (%)				
Less than high school	2891 (8.58)	2732 (8.38)	159 (14.74)	< 0.0001
High school	7509 (22.29)	7201 (22.08)	308 (28.54)	
More than high school	23,287 (69.13)	22,675 (69.54)	612 (56.72)	
Poverty income ratio, *n* (%)				
≤ 1.0	6765 (20.08)	6333 (19.42)	432 (40.04)	< 0.0001
1.0–3.0	12,967 (38.49)	12,816 (39.30)	151 (13.99)	
> 3.0	13,955 (41.43)	13,459 (41.28)	496 (45.97)	
Marriage, *n* (%)				
Divorced/separated/widowed	7366 (21.87)	6978 (21.40)	388 (35.96)	< 0.0001
Married/living with partner	20,129 (59.75)	19,663 (60.30)	466 (43.19)	
Never married	6192 (18.38)	5967 (18.30)	225 (20.85)	
Drinking status, *n* (%)				
No	9007 (26.74)	8666 (26.58)	341 (31.60)	0.002
Yes	24,680 (73.26)	23,942 (73.42)	738 (68.40)	
Smoking status, *n* (%)				
No	18,519 (54.97)	18,102 (55.51)	417 (38.65)	< 0.0001
Yes	15,168 (45.03)	14,506 (44.49)	662 (61.35)	
Diabetes mellitus, *n* (%)				
No	27,517 (81.68)	26,741 (82.01)	776 (71.92)	< 0.0001
Yes	6170 (18.32)	5867 (17.99)	303 (28.08)	
Hypertension, *n* (%)				
No	19,331 (57.38)	18,829 (57.74)	502 (46.52)	< 0.0001
Yes	14,356 (42.62)	13,779 (42.26)	577 (53.48)	
Work activity, *n* (%)				
Vigorous	1229 (4.10)	1181 (4.08)	48 (4.45)	0.52
Moderate	6756 (22.54)	6543 (22.58)	213 (19.74)	
Other	21,995 (73.37)	21,253 (73.34)	742 (68.77)	
Recreational activity, *n* (%)				
Vigorous	2281 (7.61)	2247 (7.75)	34 (3.15)	< 0.0001
Moderate	7737 (25.81)	7539 (26.02)	198 (18.35)	
Other	19,963 (66.59)	19,192 (66.23)	771 (71.46)	
Energy intake (kcals/day)	2167.27 (8.16)	2171.24 (8.20)	2035.80 (52.24)	0.01
Fiber intake (g/day)	16.91 (0.13)	16.99 (0.13)	14.24 (0.47)	< 0.0001

*Note:* Continuous variables are shown as weighted means with standard errors. Categorical variables are presented as unweighted counts along with weighted percentages.

### 3.2 Relationship Between Dietary Fiber Intake and CRD

This study employed survey‐weighted generalized linear models to evaluate the relationship of dietary fiber intake and the risk of CRD. Findings of us regarding the link of dietary fiber and CRD revealed a negative correlation, indicating that dietary fiber might decrease the risk of CRD (Table 3). The unadjusted crude model did not make any adjustments for relevant factors, while Model 1 took into account basic characteristics such as gender, race, BMI, and age. Model 2 further adjusted for education level, poverty‐income ratio, and marital status on the basis of Model 1. Model 3 adjusted for all potentially relevant factors. In the fully adjusted model, dietary fiber intake was negatively associated with CRD (odds ratios (OR) = 0.98; 95% confidence interval (95% CI): 0.97–0.99, *p* < 0.0001). In comparison with participants in the first quartile, those in the second, third, and fourth quartiles had a 17% (OR = 0.83; 95% CI: 0.70–0.97, *p* = 0.02), 22% (OR = 0.78; 95% CI: 0.65–0.92, *p* = 0.005), and 30% (OR = 0.70; 95% CI: 0.57–0.85, *p* < 0.001) lower risk of CRD, respectively, in the fully adjusted model. The strikingly similar trend was noted in the crude model, Model 1, and Model 2. The trend *p* values for all models were < 0.001, indicating a decreasing trend in CRD incidence with increasing dietary fiber intake, highlighting its protective effect against the disease. Across all models, the strongest protection and lowest risk of CRD were observed when dietary fiber intake exceeded 21.1 g per day, which corresponds to the fourth quartile. To verify our findings, we further investigated the relationship between three main sources of dietary fiber, including total fruit intake, total vegetable intake, and total grain intake, and CRD. The analysis showed that total fruit intake, total vegetable intake, and total grain intake were negatively correlated with CRD, indicating that foods rich in dietary fiber may also reduce the risk of CRD (Table S1). In the fully adjusted model, compared with participants in the first quartile, total fruit intake, total vegetable intake, and total grain intake were associated with a 28% (OR = 0.72; 95% CI: 0.61–0.86, *p* < 0.001), 37% (OR = 0.63; 95% CI: 0.51–0.77, *p* < 0.0001), and 34% (OR = 0.66; 95% CI: 0.55–0.79, *p* < 0.0001) lower risk of CRD, respectively.

**TABLE 3:** The results of a weighted multiple logistic regression analysis investigating the association between fiber intake and CRD.

Variables	Primary model		Model 1		Model 2		Model 3	
	OR (95% CI)	*p*	OR (95% CI)	*p*	OR (95% CI)	*p*	OR (95% CI)	*p*
Continuous	0.97 (0.96, 0.98)	< 0.0001	0.97 (0.97, 0.98)	< 0.0001	0.98 (0.98, 0.99)	< 0.0001	0.98 (0.97, 0.99)	< 0.0001
Fiber intake (quartile)								
Q1	Ref		Ref		Ref		Ref	
Q2	0.69 (0.59, 0.81)	< 0.0001	0.71 (0.61, 0.83)	< 0.0001	0.81 (0.69, 0.94)	0.005	0.83 (0.70, 0.97)	0.02
Q3	0.57 (0.49, 0.68)	< 0.0001	0.62 (0.52, 0.74)	< 0.0001	0.76 (0.64, 0.89)	0.001	0.78 (0.65, 0.92)	0.005
Q4	0.47 (0.40, 0.55)	< 0.0001	0.54 (0.45, 0.63)	< 0.0001	0.68 (0.58, 0.80)	< 0.0001	0.70 (0.57, 0.85)	< 0.001
*p* for trend		< 0.0001		< 0.0001		< 0.0001		< 0.001

*Note:* Primary model: adjusted for none. Model 1: The age, gender, BMI and race of participants were adjusted. Model 2: The age, gender, BMI, education level, race, PIR and marriage of participants were adjusted. Model 3: The age, gender, race, education level, BMI, PIR, marriage, energy intake, diabetes mellitus status, hypertension status, smoking status, drinking status, work activity, and recreational activity of participants were adjusted. Q1, ≤ 9.2 g/day; Q2, 9.2–14.2 g/day; Q3, 14.2–21.1 g/day; Q4, ≥ 21.1 g/day.

**FIGURE 2:**
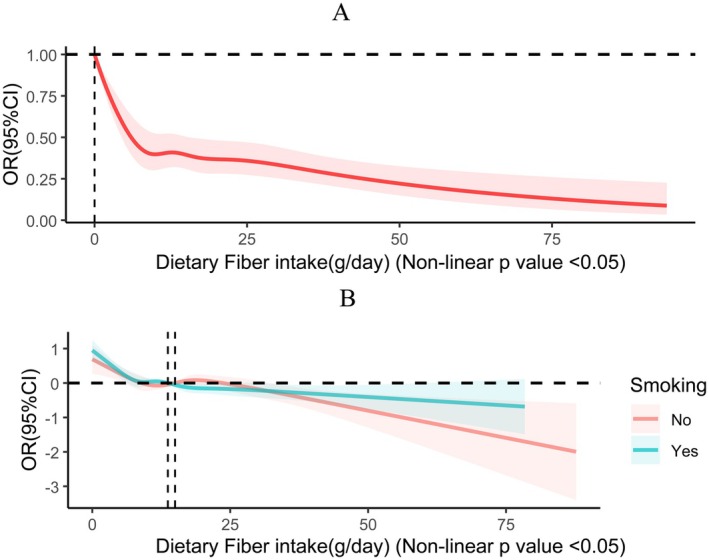
(A) RCS curves illustrating the dose–response association between fiber intake and CRD. (B) RCS curves illustrating the dose–response association between fiber intake and CRD by grouping based on smoking status. The following covariates were modulated: race, age, BMI, gender, PIR, education level, marriage, energy intake, diabetes mellitus status, hypertension status, smoking status, drinking status, work activity, and recreational activity.

Furthermore, this study made use of RCS curves to examine the dose–response correlation between the incidence of CRD and dietary fiber intake. The multivariate‐adjusted spline regression model further confirmed a nonlinear negative correlation between dietary fiber intake and CRD risk (nonlinear *p* value < 0.05), with the odds ratios (OR) curve for CRD risk taking an L‐shape as dietary fiber intake increased (Figure 2). RCS curve analysis was also conducted among subgroups stratified by smoking status, revealing a more pronounced downward trend in the OR curve with increasing dietary fiber intake in the non‐smoking group, whereas the decline was less pronounced in the smoking group. Under the completely adjusted model, all of the aforementioned analyses were conducted.

### 3.3 Relationship Between Dietary Fiber Intake and CSD

This study employed survey‐weighted generalized linear models to evaluate the relationship in which the quantity of dietary fiber that a person consumes over a certain period is associated with the risk of CSD. Our findings regarding the association between dietary fiber and CSD indicated a negative correlation, suggesting a potential reduction in CSD risk (see Table 4). The unadjusted, unmodulated crude model did not make any adjustments for relevant factors, while Model 1 considered basic characteristics such as gender, race, BMI, and age. Model 2 added education level, poverty‐income ratio, and marital status on the basis of Model 1. Model 3 adjusted for all possible relevant factors. In the fully adjusted model, dietary fiber intake was negatively associated with CSD (OR = 0.984; 95% CI: 0.969–0.999, *p* = 0.037). As a categorical variable, all four models consistently demonstrated a reduced risk of CSD with higher dietary fiber intake. Compared to patients who participated in the first quartile, those in the second, third, and fourth quartiles had a 22% (OR = 0.78; 95% CI: 0.59–1.02, *p* = 0.07), 40% (OR = 0.60; 95% CI: 0.46–0.79, *p* < 0.001), and 32% (OR = 0.68; 95% CI: 0.48–0.94, *p* = 0.02) lower risk of CSD, individually in the fully adjusted model. The same trend was observed in the raw model as well as Model 1 and Model 2. The trend *p* values for all models were < 0.004, indicating a decreasing trend in CSD incidence with increasing dietary fiber intake, underscoring its protective effect against the disease. Across all models, the strongest protection and lowest risk of CSD were observed when dietary fiber intake ranged between 14.2 and 21.1 g per day, which corresponds to the third quartile. To verify our results, we further explored the associations of total fruit intake, total vegetable intake, and total grain intake with CSD. The analysis indicated that total vegetable and grain intakes were negatively correlated with CSD (Table S2). In the fully adjusted model, compared with participants in the first quartile, total vegetable and grain intakes were associated with a 50% (OR = 0.50; 95% CI: 0.37–0.68, *p* < 0.0001) and 47% (OR = 0.53; 95% CI: 0.40–0.70, *p* < 0.0001) lower risk of CSD, respectively. However, total fruit intake was associated with a 36% lower risk of CSD only at the third quartile level.

**TABLE 4:** The results of a weighted multiple logistic regression analysis investigating the association between fiber intake and CSD.

Variables	Primary model		Model 1		Model 2		Model 3	
	OR (95% CI)	*p*	OR (95% CI)	*p*	OR (95% CI)	*p*	OR (95% CI)	*p*
Continuous	0.969 (0.956, 0.982)	< 0.0001	0.974 (0.960, 0.987)	< 0.001	0.984 (0.972, 0.996)	0.009	0.984 (0.969, 0.999)	0.037
Fiber intake (quartile)								
Q1	Ref		Ref		Ref		Ref	
Q2	0.63 (0.49, 0.81)	< 0.001	0.65 (0.51, 0.83)	< 0.001	0.75 (0.59, 0.97)	0.03	0.78 (0.59, 1.02)	0.07
Q3	0.49 (0.38, 0.62)	< 0.0001	0.52 (0.40, 0.66)	< 0.0001	0.64 (0.50, 0.82)	< 0.001	0.60 (0.46, 0.79)	< 0.001
Q4	0.47 (0.36, 0.61)	< 0.0001	0.53 (0.40, 0.69)	< 0.0001	0.69 (0.53, 0.92)	0.01	0.68 (0.48, 0.94)	0.02
*p* for trend		< 0.0001		< 0.0001		0.002		0.004

*Note:* Primary model: adjusted for none. Model 1: The age, gender, BMI and race of participants were adjusted. Model 2: The age, gender, BMI, education level, race, PIR and marriage of participants were adjusted. Model 3: The age, gender, race, education level, BMI, PIR, marriage, energy intake, diabetes mellitus status, hypertension status, smoking status, drinking status, work activity, and recreational activity of participants were adjusted. Q1, ≤ 9.2 g/day; Q2, 9.2–14.2 g/day; Q3, 14.2–21.1 g/day; Q4, ≥ 21.1 g/day.

**FIGURE 3:**
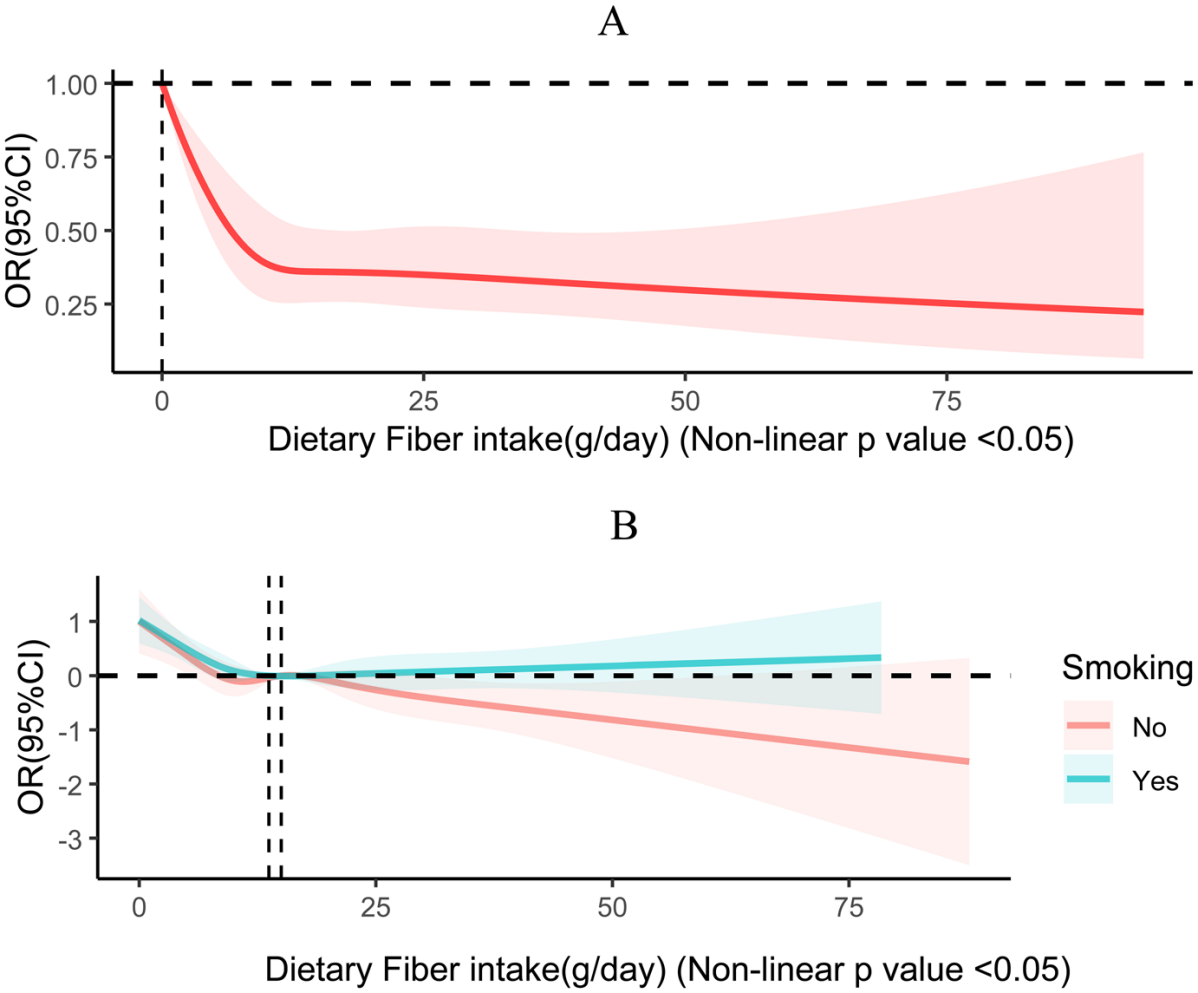
(A) RCS curves illustrating the dose–response association between fiber intake and CSD. (B) RCS curves illustrating the dose–response association between fiber intake and CSD by grouping based on smoking status. The following covariates were modulated: race, age, BMI, gender, PIR, education level, marriage, energy intake, diabetes mellitus status, hypertension status, smoking status, drinking status, work activity, and recreational activity.

Furthermore, it utilized RCS curves to explore the dose–response relationship of CSD incidence and dietary fiber intake. The multivariate‐adjusted spline regression model further confirmed a nonlinear negative correlation (nonlinear *p* value < 0.05), with the OR curve for CSD risk taking an L‐shape as dietary fiber intake increased (Figure 3). RCS curve analysis was also conducted among subgroups stratified by smoking status, revealing a more pronounced downward trend in the OR curve with increasing dietary fiber intake in the non‐smoking group, whereas the decline was less marked in the smoking group. Under the fully adjusted model, all of the aforementioned analyses were conducted.

### 3.4 Subgroup Analysis

**FIGURE 4:**
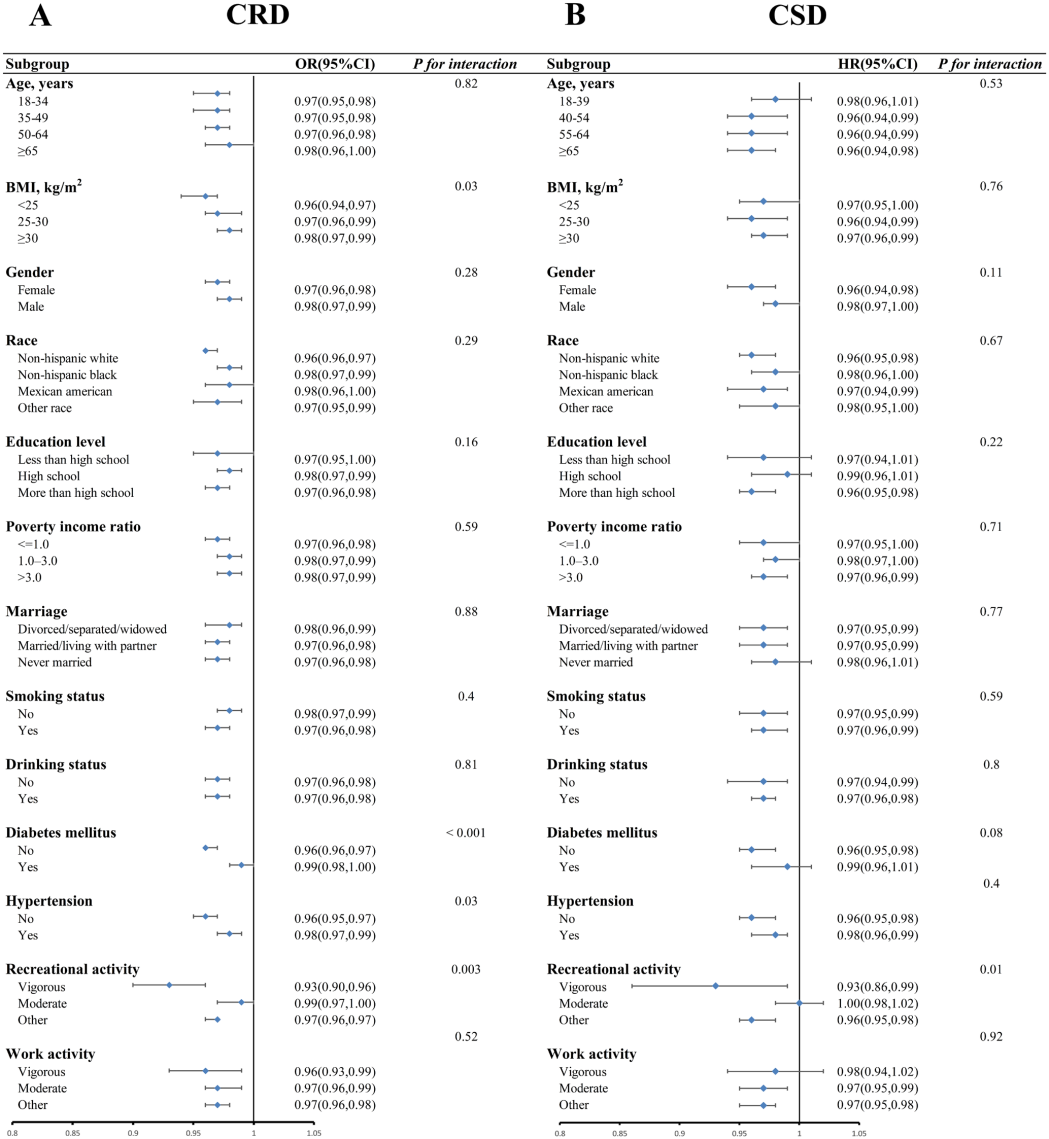
Subgroup analyses examining the association between fiber intake and CRD (A) and CSD (B) in depression.

A subgroup analysis used stratified weighted multiple regression analysis, grouping individuals based on age, BMI, gender, race, educational level, PIR, marital status, smoking status, drinking status, diabetes mellitus, hypertension, recreational activity, and work activity. This approach aimed to explore the relationship between dietary fiber intake and depression across different populations. Figure 4A presents the subgroup analysis investigating how the quantity of dietary fiber consumed over a certain period associates with CRD. Our analysis examined the interactions between dietary fiber intake and various categorical variables. Most interaction *p* values exceeded 0.05, indicating that the inverse relationship between dietary fiber intake and CRD remained consistent across these subgroups. This suggests that the protective effect of dietary fiber against CRD is not confined to specific demographic or lifestyle groups. However, a notable interaction was observed between dietary fiber intake and both diabetes and recreational activity, with interaction *p* values below 0.05. This implies that the effect of dietary fiber intake on depression may vary within these specific subgroups. Figure 4B showcases the subgroup analysis investigating the relationship between dietary fiber intake and CSD. The results indicated a consistent inverse relationship between dietary fiber intake and CSD across all demographic characteristics, lifestyle habits, and disease states, with most interaction *p* values exceeding 0.05. This further reinforces the idea that the protective effect of dietary fiber against CSD is stable and generalizable across diverse populations. In our study, we observed that the protective effect of dietary fiber intake against depression was moderated by diabetes status and recreational activity levels (interaction *p* < 0.05), suggesting that the relationship between fiber intake and depression may vary across these specific subgroups. Evidence indicates that high‐fiber diets can improve serum metabolism and mood in individuals with type 2 diabetes mellitus by modulating gut microbiota (Chen, Liu, et al. 2023). Therefore, in individuals with diabetes mellitus, the physiological stress of glycemic regulation and potential shifts in gut microbiota composition may alter fiber's mental health benefits. Regarding recreational activity, physical exercise itself has been shown to improve mental health outcomes (Pearce et al. 2022). Combined fiber intake and physical activity may synergistically enhance these benefits, potentially through shared mechanisms such as reduced inflammation and improved gut health (Moschen et al. 2012).

## 4 Discussion

This study utilized eight consecutive cycles of NHANES data (2005–2020), involving 29,980 participants, to explore the potential association between dietary fiber intake and CRD as well as CSD. The results showed that dietary fiber intake was negatively correlated with the occurrence of CRD and CSD as determined by multivariable logistic regression. Additionally, we further examined the relationship between foods high in dietary fiber and CRD and CSD. The results showed that foods rich in dietary fiber could also reduce the risk of CRD and CSD. We further investigated the dose–response relationship and found a nonlinear link between them. Subgroup analyses further confirmed the stability of this association. Our findings highlight the significance of dietary fiber intake in the prevention and management of depression. Moreover, this study also found significant differences in the association of dietary fiber intake with depression risk in subgroups stratified by smoking status by RCS curve analysis.

Our research revealed an inverse correlation between dietary fiber consumption and depression risk. Further analysis using a multivariate‐adjusted spline regression model demonstrated a nonlinear relationship between dietary fiber intake and the risk of CRD and CSD (non‐linear *p* < 0.05). The OR curves for CRD and CSD exhibited an L‐shaped pattern as dietary fiber intake increased. Notably, in all models, the protective effect against CRD was most significant when dietary fiber intake exceeded 21.1 g/day, which corresponds to the fourth quartile, and at this intake level, the lowest risk of CRD was observed. For CSD, the strongest protective effect and lowest risk were seen when dietary fiber intake was between 14.2 and 21.1 g/day, which is equivalent to the third quartile. A cross‐sectional study of patients with primary hypertension supports this view, indicating that higher dietary fiber intake serves as a protective factor against depression (Liu et al. 2021). However, with a small sample of only 459 patients from specific medical institutions, the study may have selection bias and limited representativeness. Additionally, the above‐mentioned study did not perform dose–response analyses, and its subgroup analyses were limited to basic grouping of hypertensive patients without exploring interactions with lifestyle factors. In contrast, our study included 29,980 US adults across eight consecutive two‐year cycles. Its large, nationally representative sample enhanced the statistical power. Furthermore, we utilized RCS curves to assess the relationship between dietary fiber intake and depression risk, revealing an L‐shaped nonlinear association with both CRD and CSD. Subgroup stratification analyses further showed that smoking status could influence this relationship. These nonlinear and refined subgroup analyses offer a scientific basis for precise interventions. Likewise, a study based on the Korea National Health and Nutrition Examination Survey confirmed that dietary fiber intake was inversely associated with depression risk, particularly in premenopausal women (Kim et al. 2020). However, this study also had a limited sample size and was restricted to a specific population. Notably, our study offers a distinct advantage by employing a more refined classification of depression into CRD and CSD, whereas the aforementioned study relied solely on a single threshold criterion (PHQ‐9 ≥ 10). Increasing dietary fiber intake can lower the risk of depression, underscoring the key role of adequate fiber consumption in depression prevention.

The inverse association between dietary fiber intake and depression risk may be explained by multiple mechanisms. Some studies suggest that dietary fiber may reduce depression risk via the gut‐brain axis by modulating gut microbiota composition and function (Sun et al. 2021). Among the many metabolites produced by dietary fiber, short‐chain fatty acids (SCFA), including acetic acid, propionic acid, and butyric acid, are crucial (Hays et al. 2024). Butyric acid is particularly significant because it inhibits histone deacetylase activity (Stilling et al. 2016). This inhibition helps to alleviate neuroinflammation and oxidative stress, which are both key drivers of the pathophysiology of depression. Also, SCFA can stimulate enterochromaffin cells to synthesize the 5‐hydroxytryptamine, a neurotransmitter whose reduced availability in the brain is recognized as a core feature of depressive pathogenesis (Agus et al. 2018; Gerhard et al. 2016). At the same time, the microbial metabolism of dietary fiber can promote the release of ferulic acid by bacteria with the ferulic acid esterase genes (Tomaro‐Duchesneau et al. 2012). Existing studies have shown that ferulic acid exerts anti‐depressive effects by regulating the serotonergic system (Zeni et al. 2012). Additionally, dietary fiber may be inversely associated with depression through various inflammatory markers such as the C‐reactive protein and interleukin‐6 (IL‐6), according to several studies (Kabisch et al. 2025; Shivakoti et al. 2022). A significant association has been reported in the relationship of high inflammation and an increased severity of depression. The primary mechanism through which a high dietary fiber intake may reduce inflammation is probably via the reduction of gut permeability and pH mediated by the gut microbiota, thus decreasing the production of inflammatory cytokines like IL‐6 and tumor necrosis factor‐alpha (TNF‐α) (Swann et al. 2020). Notably, our study employed RCS models to analyze the dose–response relationship between dietary fiber intake and CSD/CRD, both exhibiting L‐shaped dose–response curves. Mechanistic studies indicate that low‐to‐moderate doses (15–30 g/day) of dietary fiber may improve antidepressant outcomes by regulating gut microbiota to enhance short‐chain fatty acid production, including butyrate, thereby suppressing neuroinflammation and enhancing blood–brain barrier integrity. However, long‐chain inulin intake exceeding 30 g/day significantly increases fecal gas‐producing bacterial abundance and elevates pro‐inflammatory cytokines (IL‐6, TNF‐α), thereby reducing fiber's antidepressant efficacy. Concurrently, high‐dose inulin may overstimulate Bacteroides spp., promoting metabolic byproducts that exacerbate oxidative stress and diminish depression protective effects (Lancaster et al. 2022). This nonlinear association indicates an optimal dietary fiber intake range for antidepressant benefits. Through these multifaceted mechanisms, an important role in the prevention and treatment of depression is played by dietary fiber.

It is also noteworthy that in the RCS analysis stratified by smoking status, we observed a steeper decline in the OR curve with increasing dietary fiber intake among non‐smokers, whereas the downward trend was attenuated in current smokers. This discrepancy may stem from smoking‐induced alterations in gut microbiota composition and reductions in microbial diversity, which lead to a decrease in the ability of the gut microbiota to metabolize dietary fiber. For instance, a reduction in Bifidobacterium, a key gut bacterium that produces SCFA, may weaken its ability to ferment dietary fiber into these acids (Savin et al. 2018; Tomoda et al. 2011). Thus, smokers may not fully benefit from the protective effects of dietary fiber, leading to an indistinct downward trend in their OR curve. Furthermore, smoking induces systemic oxidative stress and the release of pro‐inflammatory cytokines like IL‐6 and TNF‐α (Csiszar et al. 2009), which may attenuate the effectiveness of dietary fiber.

However, our research also has certain inevitable limitations. First, due to the cross‐sectional nature of the study, our findings cannot be used to infer causality. Despite adjusting for multiple covariates, we cannot entirely exclude the potential influence of residual or unmeasured confounding factors. Second, since dietary fiber intake was assessed at a single time point, whereas depression develops over time, this temporal mismatch may introduce bias and limit our ability to infer the long‐term effects of dietary fiber on depression. Third, in this study, the severity of participants' depression was evaluated using the PHQ‐9 scale. Although the PHQ‐9 is a widely validated effective tool for assessing the severity of depressive symptoms, as a self‐report scale, it may be affected by recall bias and subjective reporting errors (Baryshnikov et al. 2023; Shen et al. 2022). Additionally, dietary fiber intake was assessed through 24‐h dietary recalls, which may be subject to measurement error and underreporting. These limitations should be considered when interpreting our results. Despite the above‐mentioned limitations, our study highlights the potential role of dietary fiber in reducing the prevalence of depression. However, to further confirm these findings and explore its potential application in the prevention and management of depression, it is necessary to conduct longitudinal studies and randomized controlled trials in the future.

The lack of fiber in contemporary diets can be attributed to various factors. In light of the inverse relationship of dietary fiber intake and depressive symptoms, enhancing fiber content in our diets emerges as a pivotal public health strategy. This initiative, if effectively executed, could significantly bolster metabolic health and overall well‐being, with profound implications for the psychological well‐being of coming generations.

## 5 Conclusion

In summary, our research suggests that dietary fiber intake is significantly negatively correlated with the risk of CRD and CSD, and this association remains robust after fully adjusting for confounding factors. Notably, the protective effects of dietary fiber are more significant in non‐smokers, while smoking may attenuate these effects. This study demonstrates that at the public health level, promoting increased dietary fiber intake along with enhanced smoking cessation measures holds dual significance for depression prevention. Future studies should focus on validating the causal relationships between dietary fiber and CRD as well as CSD through prospective cohort studies and randomized controlled trials, evaluating the clinical efficacy of dietary fiber interventions in depression management, and developing precision‐based nutritional strategies tailored to populations with varying smoking statuses.

## Author Contributions

**Siran Lai:** conceptualization (lead), data curation (lead), formal analysis (lead), methodology (lead), supervision (equal), visualization (lead), writing – original draft (lead), writing – review and editing (equal). **Yuning Zeng:** formal analysis (equal), software (lead), validation (lead), writing – review and editing (lead). **Tianyi Li:** data curation (lead), investigation (lead), resources (supporting), writing – review and editing (equal). **Yue Li:** data curation (equal), investigation (equal), resources (lead), writing – review and editing (equal). **Yue An:** methodology (equal), validation (equal), writing – review and editing (equal). **Xueren Ouyang:** conceptualization (lead), project administration (lead), resources (lead), supervision (lead), writing – review and editing (lead).

## Ethics Statement

Ethical approval was obtained from the National Center for Health Statistics Ethics Review Board.

## Consent

All participants signed the informed consent.

## Conflicts of Interest

The authors declare no conflicts of interest.

## Supporting information


Data S1.

## Data Availability

All relevant data and material of this study are available from the corresponding authors upon request.
