# Supplementary material for: The Association Between Dietary Fiber Intake and Depression Among US Adults: A Cross‐Sectional Study Based on NHANES Data From 2005 to 2020
Source: Food Sci Nutr. 2025 Jul 16;13(7):e70605. doi: 10.1002/fsn3.70605 (PMC12267881; doi:10.1002/fsn3.70605)
Supplement: Supplementary file 1 — Data S1. [file FSN3-13-e70605-s001.docx]

**The Association between Dietary Fiber Intake and Depression among US Adults: A Cross-sectional Study Based on NHANES Data from 2005 to 2020**

**Running title：Fiber Intake-Depression Association: NHANES Study**

Siran Lai1; Yuning Zeng; Tianyi Li; Yue Li; Yue An; Xueren Ouyang*

Table S1 The results of a weighted multiple logistic regression analysis investigating the association between total fruits intake, total vegetables intake, total grains intake, and CRD.

| Variables | Primary model | | Model 1 | | Model 2 | | | Model 3 | |
| --- | --- | --- | --- | --- | --- | --- | --- | --- | --- |
|  | OR (95% CI) | *P* value | OR (95% CI) | *P* value | OR (95% CI) | *P* value | | OR (95% CI) | *P* value |
| Continuous | 0.86(0.80,0.93) | <0.001 | 0.88(0.81,0.95) | <0.001 | 0.91(0.85,0.97) | 0.01 | 0.94(0.88,1.01) | | 0.07 |
| Total fruits intake (quartile) | | | | | | | | | |
| Q1 | Reference |  | Reference |  | Reference |  | | Reference |  |
| Q2 | 0.73(0.61,0.86) | <0.001 | 0.71(0.59,0.84) | <0.001 | 0.83(0.69,0.99) | 0.04 | | 0.88(0.73,1.06) | 0.17 |
| Q3 | 0.55(0.47,0.65) | <0.0001 | 0.55(0.46,0.65) | <0.0001 | 0.66(0.55,0.78) | <0.0001 | | 0.72(0.61,0.86) | <0.001 |
| Q4 | 0.57(0.47,0.69) | <0.0001 | 0.59(0.48,0.72) | <0.0001 | 0.69(0.56,0.85) | <0.001 | | 0.79(0.63,0.98) | 0.03 |
| *P* for trend |  | <0.0001 |  | <0.0001 |  | <0.0001 | |  | 0.01 |
| Continuous | 0.81(0.76,0.87) | <0.0001 | 0.83(0.78,0.89) | <0.0001 | 0.89(0.84,0.94) | <0.001 | | 0.89(0.83,0.95) | <0.001 |
| Total vegetables intake (quartile) | | | | | | | | | |
| Q1 | Reference |  | Reference |  | Reference |  | | Reference |  |
| Q2 | 0.75(0.64,0.87) | <0.001 | 0.75(0.63,0.88) | <0.001 | 0.82(0.70,0.96) | 0.02 | | 0.83(0.71,0.98) | 0.03 |
| Q3 | 0.59(0.50,0.70) | <0.0001 | 0.62(0.52,0.73) | <0.0001 | 0.74(0.63,0.87) | <0.001 | | 0.75(0.63,0.90) | 0.002 |
| Q4 | 0.47(0.40,0.56) | <0.0001 | 0.50(0.42,0.60) | <0.0001 | 0.62(0.52,0.74) | <0.0001 | | 0.63(0.51,0.77) | <0.0001 |
| *P* for trend |  | <0.0001 |  | <0.0001 |  | <0.0001 | |  | <0.0001 |
| Continuous | 0.96(0.94,0.98) | <0.0001 | 0.97(0.95,0.99) | 0.001 | 0.98(0.96,0.99) | 0.01 | | 0.97(0.95,0.99) | 0.002 |
| Total grains intake (quartile) | | | | | | | | | |
| Q1 | Reference |  | Reference |  | Reference |  | | Reference |  |
| Q2 | 0.80(0.67,0.95) | 0.01 | 0.81(0.68,0.96) | 0.02 | 0.84(0.71,1.00) | 0.05 | | 0.83(0.70,1.00) | 0.05 |
| Q3 | 0.68(0.58,0.79) | <0.0001 | 0.71(0.60,0.83) | <0.0001 | 0.75(0.64,0.88) | <0.001 | | 0.74(0.63,0.87) | <0.001 |
| Q4 | 0.59(0.51,0.70) | <0.0001 | 0.65(0.55,0.77) | <0.0001 | 0.71(0.60,0.84) | <0.0001 | | 0.66(0.55,0.79) | <0.0001 |
| *P* for trend |  | <0.0001 |  | <0.0001 |  | <0.0001 | |  | <0.0001 |

Primary model: adjusted for none.

Model 1: The age, gender, BMI and race of participants were adjusted.

Model 2: The age, gender, BMI, education level, race, PIR and marriage of participants were adjusted.

Model 3: The age, gender, race, education level, BMI, PIR, marriage, energy intake, diabetes mellitus status, hypertension status, smoking status, drinking status, work activity, and recreational activity of participants were adjusted.

Total fruits intake: Q1,≤0 cup; Q2,0–0.44 cup; Q3, 0.44–1.46 cup; Q4,≥ 1.46 cup.

Total grains intake: Q1,≤3.66 oz; Q2, 3.66–5.77 oz; Q3, 5.77–8.58 oz; Q4,≥ 8.58 oz.

Total vegetables intake: Q1,≤0.56 cup; Q2,0.56–1.18 cup; Q3, 1.18–2.04 cup; Q4,≥ 2.04 cup.

Table S2 The results of a weighted multiple logistic regression analysis investigating the association between total fruits intake, total vegetables intake, total grains intake, and CSD.

| Variables | Primary model | | Model 1 | | Model 2 | | | Model 3 | |
| --- | --- | --- | --- | --- | --- | --- | --- | --- | --- |
|  | OR (95% CI) | *P* value | OR (95% CI) | *P* value | OR (95% CI) | *P* value | | OR (95% CI) | *P* value |
| Continuous | 0.92(0.80,1.04) | 0.18 | 0.93(0.82,1.06) | 0.29 | 0.96(0.86,1.08) | 0.50 | 1.00(0.90,1.11) | | 0.96 |
| Total fruits intake (quartile) | | | | | | | | | |
| Q1 | Reference |  | Reference |  | Reference |  | | Reference |  |
| Q2 | 0.69(0.54,0.90) | 0.01 | 0.67(0.51,0.87) | 0.003 | 0.80(0.61,1.04) | 0.10 | | 0.84(0.64,1.11) | 0.22 |
| Q3 | 0.49(0.38,0.62) | <0.0001 | 0.48(0.37,0.60) | <0.0001 | 0.58(0.46,0.74) | <0.0001 | | 0.64(0.50,0.81) | <0.001 |
| Q4 | 0.58(0.42,0.80) | 0.001 | 0.59(0.43,0.82) | 0.002 | 0.71(0.51,1.00) | 0.05 | | 0.81(0.57,1.15) | 0.23 |
| *P* for trend |  | <0.0001 |  | <0.001 |  | 0.01 | |  | 0.07 |
| Continuous | 0.76(0.67,0.86) | <0.0001 | 0.78(0.69,0.87) | <0.0001 | 0.84(0.75,0.93) | 0.002 | | 0.84(0.75,0.94) | 0.002 |
| Total vegetables intake (quartile) | | | | | | | | | |
| Q1 | Reference |  | Reference |  | Reference |  | | Reference |  |
| Q2 | 0.77(0.59,1.01) | 0.06 | 0.77(0.58,1.01) | 0.06 | 0.85(0.65,1.12) | 0.25 | | 0.87(0.66,1.15) | 0.31 |
| Q3 | 0.56(0.43,0.73) | <0.0001 | 0.58(0.44,0.75) | <0.0001 | 0.71(0.55,0.92) | 0.01 | | 0.72(0.54,0.95) | 0.02 |
| Q4 | 0.38(0.28,0.51) | <0.0001 | 0.40(0.30,0.53) | <0.0001 | 0.50(0.37,0.66) | <0.0001 | | 0.50(0.37,0.68) | <0.0001 |
| *P* for trend |  | <0.0001 |  | <0.0001 |  | <0.0001 | |  | <0.0001 |
| Continuous | 0.95(0.93,0.98) | 0.001 | 0.96(0.94,0.99) | 0.01 | 0.97(0.94,1.00) | 0.04 | | 0.96(0.94,0.99) | 0.01 |
| Total grains intake (quartile) | | | | | | | | | |
| Q1 | Reference |  | Reference |  | Reference |  | | Reference |  |
| Q2 | 0.80(0.63,1.00) | 0.05 | 0.81(0.64,1.02) | 0.07 | 0.85(0.67,1.07) | 0.16 | | 0.84(0.66,1.07) | 0.15 |
| Q3 | 0.48(0.37,0.63) | <0.0001 | 0.50(0.39,0.66) | <0.0001 | 0.54(0.41,0.70) | <0.0001 | | 0.53(0.40,0.70) | <0.0001 |
| Q4 | 0.56(0.45,0.71) | <0.0001 | 0.62(0.48,0.79) | <0.001 | 0.68(0.53,0.88) | 0.003 | | 0.63(0.48,0.82) | <0.001 |
| *P* for trend |  | <0.0001 |  | <0.0001 |  | <0.001 | |  | <0.0001 |

Primary model: adjusted for none.

Model 1: The age, gender, BMI and race of participants were adjusted.

Model 2: The age, gender, BMI, education level, race, PIR and marriage of participants were adjusted.

Model 3: The age, gender, race, education level, BMI, PIR, marriage, energy intake, diabetes mellitus status, hypertension status, smoking status, drinking status, work activity, and recreational activity of participants were adjusted.

Total fruits intake: Q1,≤0 cup; Q2,0–0.44 cup; Q3, 0.44–1.46 cup; Q4,≥ 1.46 cup.

Total grains intake: Q1,≤3.66 oz; Q2, 3.66–5.77 oz; Q3, 5.77–8.58 oz; Q4,≥ 8.58 oz.

Total vegetables intake: Q1,≤0.56 cup; Q2,0.56–1.18 cup; Q3, 1.18–2.04 cup; Q4,≥ 2.04 cup.
